# *Propionibacterium acnes* Enhances the Immunogenicity of HIVBr18 Human Immunodeficiency Virus-1 Vaccine

**DOI:** 10.3389/fimmu.2018.00177

**Published:** 2018-02-07

**Authors:** Daniela Teixeira, Mayari Eika Ishimura, Juliana de Souza Apostólico, Jacqueline Miyuki Viel, Victor Cabelho Passarelli, Edecio Cunha-Neto, Daniela Santoro Rosa, Ieda Maria Longo-Maugéri

**Affiliations:** ^1^Division of Immunology, Department of Microbiology, Immunology and Parasitology, Federal University of São Paulo, São Paulo, Brazil; ^2^Laboratory of Clinical Immunology and Allergy-LIM60, School of Medicine, University of São Paulo, São Paulo, Brazil

**Keywords:** *Propionibacterium acnes*, adjuvant, human immunodeficiency virus-1, DNA vaccine, CD4^+^ T cell, immunomodulation

## Abstract

Immunization of BALB/c mice with HIVBr18, a DNA vaccine containing 18 CD4^+^ T cell epitopes from human immunodeficiency virus (HIV), induced specific CD4^+^ and CD8^+^ T cell responses in a broad, polyfunctional and persistent manner. With the aim of increasing the immunogenicity of this vaccine, the effect of *Propionibacterium acnes* as an adjuvant was evaluated. The adjuvant effects of this bacterium have been extensively demonstrated in both experimental and clinical settings. Herein, administration of two doses of HIVBr18, in the presence of *P. acnes*, increased the proliferation of HIV-1-specific CD4^+^ and CD8^+^ T lymphocytes, the polyfunctional profile of CD4^+^ T cells, the production of IFN-γ, and the number of recognized vaccine-encoded peptides. One of the bacterial components responsible for most of the adjuvant effects observed was a soluble polysaccharide extracted from the *P. acnes* cell wall. Furthermore, within 10 weeks after immunization, the proliferation of specific T cells and production of IFN-γ were maintained when the whole bacterium was administered, demonstrating a greater effect on the longevity of the immune response by *P. acnes*. Even with fewer immunization doses, *P. acnes* was found to be a potent adjuvant capable of potentiating the effects of the HIVBr18 vaccine. Therefore, *P. acnes* may be a potential adjuvant to aid this vaccine in inducing immunity or for therapeutic use.

## Introduction

Human immunodeficiency virus (HIV) was discovered and characterized more than thirty years ago (1, 2), and the epidemic it causes is still a significant public health problem. Despite favorable changes in the incidence of HIV in many countries, data from the World Health Organization estimated that more than 36.7 million people worldwide were living with HIV, and 1.1 million lost their lives to acquired immune deficiency syndrome (AIDS) in 2015 (3).

Adaptive immunity against HIV-1 infection is mediated by specific CD4^+^ and CD8^+^ lymphocytes and by neutralizing and non-neutralizing antibodies (4, 5). The contribution of cytotoxic T lymphocytes (CTLs) in the control of viremia has been demonstrated (6). However, although CTLs positively influence the progression of clinical diseases (7, 8), CTLs alone are not sufficient to control disease (9).

CD4^+^ T lymphocytes, whose mass destruction by infection facilitates the development of the main symptoms of AIDS, can also play a protective role. These cells assist the induction and maintenance of CTL responses, the differentiation of B lymphocytes into plasma cells, and the expansion of memory B cells (10–12). Proliferation of HIV-1-specific CD4^+^ T lymphocytes has been shown to be related to decreased viremia by controlling HIV-1 replication (13), and polyfunctional activity of these cells in the mucosal region, as well as the presence of HIV-specific cytotoxic CD4^+^ T lymphocytes, may be beneficial during HIV-1 progression (14–18). Moreover, depletion of CD4^+^ T cells has been shown to result in reduced protection against SIV challenge in SIV-vaccinated non-human primates (19). Due to the importance of the cellular immune response against HIV-1 infection, the development of vaccine candidates targeting conserved T lymphocyte epitopes, especially of CD4^+^ T cells, has become relevant (20, 21).

In previous reports, our group scanned the entire consensus proteome of HIV-1 subtype B with an algorithm called TEPITOPE, and we mapped 18 HIV-1-specific CD4^+^ T cell epitopes that were able to promiscuously bind to different HLA molecules (DR, DQ, and DP) (22). Peripheral blood mononuclear cells from more than 90% of evaluated HIV patients were able to recognize these peptides. Following this analysis, DNA vaccine HIVBr18, encoding such epitopes, was produced (23, 24). Immunization of mice transgenic for common HLA class II molecules with HIVBr18 led to extensive CD4^+^ and CD8^+^ T lymphocyte responses that targeted 16 of 18 encoded epitopes and exhibited a persistent and polyfunctional profile (23). These studies suggested that such a vaccine could have broad coverage in the human population that could in turn recognize multiple HIV-1 peptides.

DNA vaccines have several advantages over recombinant viral vaccines, including being easier to access and cheaper to prepare and having a quicker mode of deployment in case of an emerging infection epidemic. However, the suboptimal immunogenicity of DNA vaccines in humans is a significant drawback and is probably due to inefficient transfection (25). Several approaches have been used to enhance their immunogenicity and enable efficient use in humans (26–28). Indeed, an ideal adjuvant for DNA vaccines should be capable of improving DNA uptake, gene expression, antigen processing and presentation and of modulating both the innate and adaptive immune responses. Some adjuvants are currently being used in clinical trials involving combined use with DNA vaccines, such as glucopyranosyl lipid adjuvant-aqueous formulation (29).

*Propionibacterium acnes* is a Gram-positive anaerobic bacillus that belongs to the normal cutaneous microbiota (30) and has beneficial immunomodulatory activity when used as a heat- or phenol-killed suspension. Since it is extensively used as an adjuvant in clinical trials, *P. acnes* can be a suitable candidate for vaccine approaches in humans (31, 32). Among its main biological activities, *P. acnes* promotes macrophage activation (33, 34), exhibits tumoricidal activity (34–39) and induces an adjuvant effect on antibody responses (40, 41), which altogether seem to explain the increase in pathogen resistance observed after intraperitoneal or subcutaneous treatment with this bacterium (42–47). The mechanisms responsible for the modulating effects of *P. acnes* on both innate and acquired immunity are mediated by proinflammatory cytokines, which are induced by treatment with this adjuvant in a manner dependent on TLR2, TLR9, and MyD88 (48–51). Due to this cytokine pattern, the killed *P. acnes* suspension has been used as a Th1 response inducer (52–54).

To investigate which component from *P. acnes* is related to the effects observed with the whole bacterium treatment, a cell wall polysaccharide (PS) purified from this bacterium was characterized by our group (55), and in different models, PS was shown to induce similar effects as those generated by the total bacterial suspension. The heat-killed *P. acnes* suspension and PS were both able to enhance an antibody response to a *Trypanosoma cruzi* DNA vaccine in mice (41), increase the number and tumoricidal activity of peritoneal macrophages (38), and enhance the number and maturation of dendritic cells (DCs) (56). Furthermore, we revealed that *P. acnes* and PS could not only direct a typical Th1 response but also enhance the elicited Th2 pattern in a murine model of type I hypersensitivity reaction (55, 57). Thus, these findings indicate that PS may be one of the leading *P. acnes* components related to its beneficial effects.

The adjuvant effect of *P. acnes* on these experimental models has led to the conclusion that its modulation of the immune response probably occurs by direct action on antigen-presenting cells (APCs) (58). Indeed, the effect of *P. acnes* in recruiting and maturing DCs (52, 56) and its immunomodulation of the activation status of APCs, such as B lymphocytes, macrophages and DCs, which are responsible for T cell direction, have been previously demonstrated (58).

As *P. acnes* has been previously shown to increase the immunogenicity of a DNA vaccine against *T. cruzi* in mice (41), herein, we investigated whether its association could also improve the immunogenicity of HIVBr18 immunization in BALB/c animals. Moreover, we examined whether PS could be related to one of the mechanisms by which *P. acnes* may function when associated with this vaccine. Due to its approved use in humans, previous use in clinical trials and commercial use in immunotherapy for immunosuppressed patients, *P. acnes* may be a promising adjuvant for increasing the immunogenicity of the DNA vaccine HIVBr18 in humans.

In addition, other adjuvants, such as bupivacaine (59) and a plasmid encoding GM-CSF (60), have also been simultaneously used with HIVBr18 in experimental models. However, although both induced some adjuvant effect, neither bupivacaine nor GM-CSF produced the immunomodulatory effect elicited by *P. acnes*, namely, increasing the magnitude and amplifying the recognition of HIVBr18 epitopes.

## Materials and Methods

### Animals

Eight-week-old female BALB/c mice (H-2^d^) were housed and handled under specific pathogen-free conditions at the animal care facilities of the Immunology Division of Federal University of São Paulo. Animal handling was conducted in strict compliance with the National Institutes of Health Guide for the Care Use of Laboratory Animals and the Brazilian National Law (11.794/2008). This study was approved by the Institutional Animal Care and Use Committee of the Federal University of São Paulo (permit number 4836281114).

### Adjuvants

#### Heat-Killed *P. acnes* Suspension

*Propionibacterium acnes* strain was obtained from Adolfo Lutz Institute, SP, Brazil. After three days in culture using anaerobic medium (Hemobac, Probac, SP, Brazil) at 37°C, bacteria were washed three times at 2,000 *g* for 30 min and then resuspended in saline. The bacterial suspension was autoclaved at 120°C for 20 min, and protein concentration was determined using the Bradford method (61) and was then used to establish individual doses for immunization.

#### *P. acnes* Soluble PS Fraction

Polysaccharide was obtained by phenol-extraction and ethanol precipitation, as previously described by our group (55) and based on Palmer and Gerlough protocol for PS extraction (62). The Bradford method (61) was used to confirm PS purity with the absence of proteins, and the carbohydrate concentration was determined using the Dubois method (63).

### DNA Vaccine

The DNA vaccine HIVBr18, which was previously described (23, 24), was used in this study. This construct was designed to encode sequences for 18 CD4^+^ epitopes derived from HIV-1 subtype B consensus sequence (22): gag1 to gag4, pol1 to pol3, env1 to env5, rev, vpr2 and vpr3, vif2, vpu, and nef. Large-scale purifications of the empty vector pVAX1 and pVAX-HIVBr18 plasmid were performed using an EndoFree Plasmid Giga Kit (Qiagen) according to the manufacturer’s instructions. The obtained DNA was evaluated for yield and purity by spectrophotometry at 260 nm and by endonuclease digestion with *HindIII* and *XhoI*.

### Peptides

The 18 epitopes encoded by the DNA vaccine HIVBr18 were synthesized by solid-phase technology with amidation of the C-terminal carboxyl group (Biomatik). Peptide purity was defined as above 90%.

### Immunization Schedule

Six mice per group were intramuscularly injected with two doses of 100 µg of plasmid pVAX-HIVBr18 or empty vector pVAX1 per dose (1 µg/µl, in saline) and 50 µg for each quadriceps. Doses were administered at an interval of 2 weeks, and each dose was mixed with adjuvants (70 µg of protein of the heat-killed *P. acnes* suspension or 25 µg of PS) or with 0.9% saline. Mice were euthanized, and splenocytes were collected 2 or 10 weeks after the last immunization. Experiments were repeated up to three times.

### Cell Isolation for Immune Assays

Single-cell suspensions of splenocytes from the immunized groups were obtained 2 or 10 weeks after the last immunization by pooling samples from the animals in each group; however, for the cytotoxicity assay, each animal was individually analyzed. R10 medium (RPMI-1640 supplemented with 10% fetal bovine serum, 2 mM l-glutamine, 1 mM sodium pyruvate, 1% vitamin solution, 1% nonessential amino acids, penicillin/streptomycin, 28 mM HEPES, 23.8 mM sodium bicarbonate, and 55 mM 2-mercaptoethanol, Gibco) was used to resuspend cells, and cell viability was determined using trypan blue (Gibco).

### IFN-γ ELISpot Assay

After *in vitro* stimulation of splenocytes from immunized mice (5 × 10^5^ cells/well) with 5 µM of 18 HIV-1 peptides (either pooled or individual), an ELISpot assay was performed using a mouse IFN-γ ELISpot Ready-SET-Go! Kit (eBioscience) according to the manufacturer’s instructions. Expression of spot-forming units (SFU/10^6^ cells) was used to refer to the number of antigen-specific T lymphocytes and was calculated after subtracting values obtained for the unstimulated condition, and 15 SFU/10^6^ cells was considered as a cutoff.

### Cytometric Bead Array

Splenocytes from immunized mice were cultured (1 × 10^6^ cells/well) with 5 µM of pooled HIV-1 peptides for five days under an atmosphere of 5% CO_2_ at 37°C. Supernatants were harvested, and cytokines were detected using a Mouse Th1/Th2/Th17 Cytometric Bead Array Kit (BD) according to the manufacturer’s instruction.

### Proliferation Assay

To assess whether T lymphocytes from immunized animals proliferated in response to HIV-1-specific antigen, spleen cells were stained with 1.25 µM of carboxyfluorescein succinimidyl ester (CFSE) and cultured (triplicates of 5 × 10^5^ cells/well) with 5 µM of pool of HIV-1 peptides or medium (R10) alone for five days under an atmosphere of 5% CO_2_ at 37°C. Cells were then collected and labeled for 30 min at 4°C with CD3 PE (145-2C11), CD4 PerCP (RM4-5), and CD8 APC (53-6.7) antimouse monoclonal antibodies (BD) for proliferation evaluation. After washing, samples were analyzed using a FACSCanto™ II (BD) with appropriate compensation controls (single stained beads, CompBeads (BD), or CFSE-single stained cells). The frequency of proliferating T lymphocyte subpopulations (CFSE^low^) among CD4^+^ CD3^+^ or CD8^+^ CD3^+^ cells (Figure S1 in Supplementary Material) was determined using FlowJo software, version 9.7.6 (Tree Star). The HIV-specific population was determined by estimating the number of CFSE^low^ cells after HIV-1-specific stimulus and after subtracting values obtained for the unstimulated condition.

### Detection of Intracellular Cytokine Production

To evaluate cytokine production from proliferating cells, spleen cells from immunized animals were stained with CFSE and cultured with 5 µM of the pool of HIV-1 peptides or medium (R10) alone as described above. However, for the last 12 h, cells were restimulated with the same antigen, 2 µg/mL of anti-CD28 (eBioscience) and Brefeldin A-GolgiPlug™ (BD). In the end, cells were collected and labeled with CD3 APC-Cy7 (145-2C11), CD4 PerCP (RM4-5), and CD8 Pacific Blue (53-6.7) antimouse monoclonal antibodies (BD) for 30 min at 4°C. Cells were then fixed and permeabilized using a Cytofix/Cytoperm™ kit (BD) and then labeled with IL-2 PE (JES6-5H4), TNF-α PE-Cy7 (MP6-XT22) and IFN-γ APC (XMG1.2) antimouse monoclonal antibodies (BD) for 30 min at 4°C. After washing, samples were analyzed using a FACSCanto™ II (BD). The frequency of proliferating T cells that also produced each of the cytokines were determined by FlowJo software (Figure S1 in Supplementary Material). In addition, a Boolean gating platform was used to create all possible combinations of cytokine-producing cells.

### Cytotoxicity Assay

Two groups of three to five mice were immunized with two doses of DNA HIVBr18 as described above, such that one group received no adjuvant, while the other received 70 µg of *P. acnes* at the first dose only. Two weeks after the last immunization, the cell line RAW 264.7 (mouse leukemic monocyte macrophage) was cultured in six-well plates (3 × 10^5^ cells/well) under an atmosphere of 5% CO_2_ at 37°C and maintained in R10 for 24 h. After this period, the cells were transfected with 2 µL of FuGENE^®^ HD Transfection Reagent (Promega) and 3 µg of pVAX-HIVBr18 or 3 µg of empty vector pVAX1 in Macrophage Medium (Gibco). After a 6-h incubation, R10 without antibiotic was added to each well. The next day, the culture medium was changed to R10 with [methyl-^3^H] thymidine (5 μCi/mL, Amersham Biosciences). After 24 h, transfected RAW 264.7 cells were collected after dissociation with 1% PBS-EDTA, and cellular concentration and viability were determined using trypan blue. Then, 1 × 10^4^ of both transfected cells were cocultured with splenocytes from immunized mice at a 1:50 ratio of target to effector cells in 96-well round bottom plates to detect cytotoxicity (E) against target cells expressing or not expressing the HIV-1 antigen (transfected cells). Cells were incubated for 3.5 h under an atmosphere of 5% CO_2_ at 37°C and were then collected using a cell harvester (PerkinElmer). A β-counter (MicroBeta^2^ LumiJET—PerkinElmer) was used to measure radioactivity in counts per minute (CPM). Spontaneous lysis (S) was determined by culturing transfected cells alone in R10. The lysis percentage was calculated using the following equation:

%Lysis=[(Scpm−Ecpm)/Scpm]×100.

The specific lysis percentage was determined by subtracting the lysis value obtained when the target cell was transfected with pVAX-HIVBr18 (specific) from the lysis value obtained when the target cell was transfected with empty pVAX1 (unspecific).

### Data Analysis

Significant differences between vaccinated groups were calculated by one-way analysis of variance followed by Tukey’s Multiple Comparison Test using GraphPad Prism software.

## Results

### Broad, Specific, and Persistent T Cell Responses after HIVBr18 Immunization Coadministered with *P. acnes*

**Figure 1:**
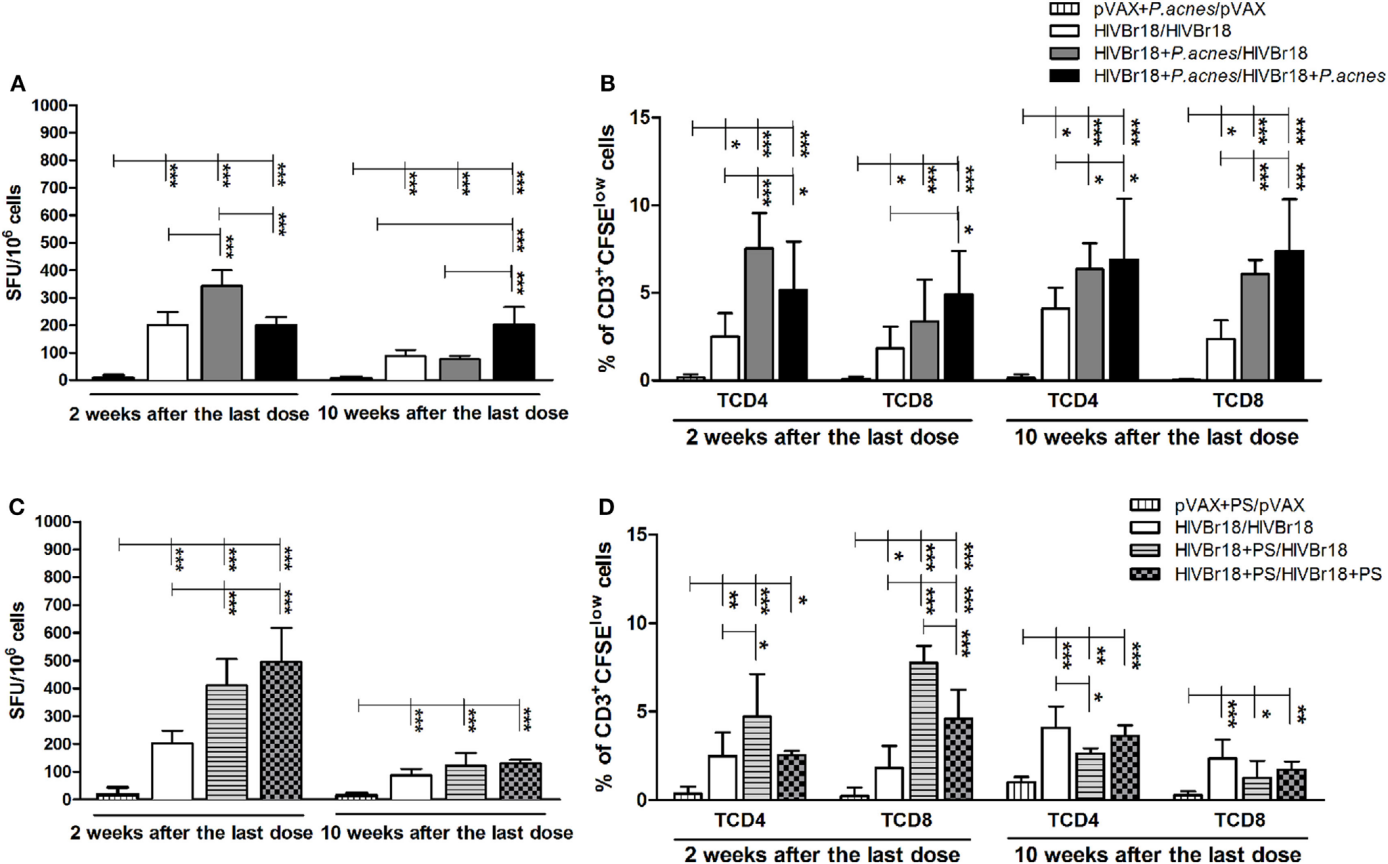
The magnitude of specific cellular responses to the pool of human immunodeficiency virus (HIV)-1 peptides induced by HIVBr18 was potentiated and persisted when mice were immunized in the presence of *Propionibacterium acnes*. Two or 10 weeks after the last immunization with empty vector pVAX1 or HIVBr18, with or without *P. acnes* **(A,B)** or polysaccharide **(C,D)**, splenocytes were either cultured for 18 h with pooled HIV-1 peptides and evaluated for IFN-γ production by ELISpot assay **(A,C)** or stained with carboxyfluorescein succinimidyl ester (CFSE) (1.25 µM) before culturing with the pooled peptides for 5 days **(B,D)**. Antigen-specific proliferation was defined by CFSE dilution in gated CD3^+^CD4^+^ and CD3^+^CD8^+^ cells by flow cytometry. SFU, spot-forming units. Cutoff = 15 SFU/10^6^ cells. Results are presented as values (mean ± SD) for each group from three independent experiments. *p < 0.05; **p < 0.001; ***p < 0.0001.

To evaluate the adjuvant effect of *P. acnes* and whether its soluble PS fraction mediates bacterium action on the immunogenicity of HIVBr18, mice were administered the DNA vaccine alone or with the adjuvant in the first or in both doses. After 2 weeks, the cellular immune response was assessed, and compared to HIVBr18 alone (201 SFU/10^6^ cells) and *P. acnes* in both doses (201.8 SFU/10^6^ cells), addition of *P. acnes* to only the first dose was found to significantly increase the number of IFN-γ producing cells (343.1 SFU/10^6^ cells) (Figure 1A). In contrast, 10 weeks after the last dose, mice that received HIVBr18 with *P. acnes* in both doses expressed a higher number of IFN-γ-producing cells (202.5 SFU/10^6^ cells) than the other mice (87.3 SFU/10^6^ cells for mice that did not receive adjuvant, and 76.4 SFU/10^6^ cells for mice that received *P. acnes* only in the first dose, Figure 1A). As expected, in animals immunized with empty vector pVAX1 in the presence of *P. acnes*, the number of these cells was lower than the cutoff value.

Human immunodeficiency virus-specific T cell proliferation analysis (Figure 1B) demonstrated that, 2 weeks postimmunization with HIVBr18, the presence of *P. acnes* in the first or in both doses enhanced the frequency of CD4^+^ T lymphocytes to a greater degree than the absence of adjuvant (7.53, 5.16, and 2.51%, respectively). Although the frequency of CD8^+^ proliferating T cells was also higher in the groups that received *P. acnes*, the group that received two doses of this adjuvant showed a significantly higher frequency (4.9%) of proliferating cells than the group that received *P. acnes* with the first dose (3.85%) and the group that received no adjuvant (1.83%). Even 10 weeks after the last immunization, the presence of *P. acnes* in the first or in both doses induced a higher frequency of proliferation of HIV-1-specific CD4^+^ (6.35 and 6.69%, respectively) and CD8^+^ (6.07 and 7.38%, respectively) T cells than the group that received no adjuvant (4.10% for CD4^+^ and 2.37% for CD8^+^ T cells). As expected, immunization with pVAX1 did not generate detectable HIV-1-specific T cell proliferation.

The addition of PS to HIVBr18 also increased the magnitude of IFN-γ production (Figure 1C) 2 weeks after the last immunization when administered in the first dose (411 SFU/10^6^ cells) and in both doses (495.6 SFU/10^6^ cells). As expected, in pVAX1-expressing mice that were immunized in the presence of PS, the number of IFN-γ-producing cells was below the cutoff value. Administration of PS in the first dose also induced higher HIV-1-specific CD4^+^ T cell proliferation (4.73%) than the absence of adjuvant (2.51%) and administration of PS in both doses (2.56%) (Figure 1D). Regarding the CD8^+^ T cell component, the PS adjuvant effect was more prominent, such that administration of PS increased the frequency of these HIV-1-specific cells in both groups of animals that received PS (7.76 and 4.6%, Figure 1D). As expected, spleen cells from pVAX1-immunized mice presented low levels of proliferation. However, different from *P. acnes* treatment, PS administration in the first or in both doses could not maintain the magnitude of the cellular response for 10 weeks, neither on IFN-γ producing cells (121.7 and 129.3 SFU/10^6^ cells, Figure 1C) nor on the frequency of proliferating (Figure 1D) CD4^+^ (2.63 and 3.63%, respectively) and CD8^+^ T cells (1.27 and 1.73%, respectively).

**Figure 2:**
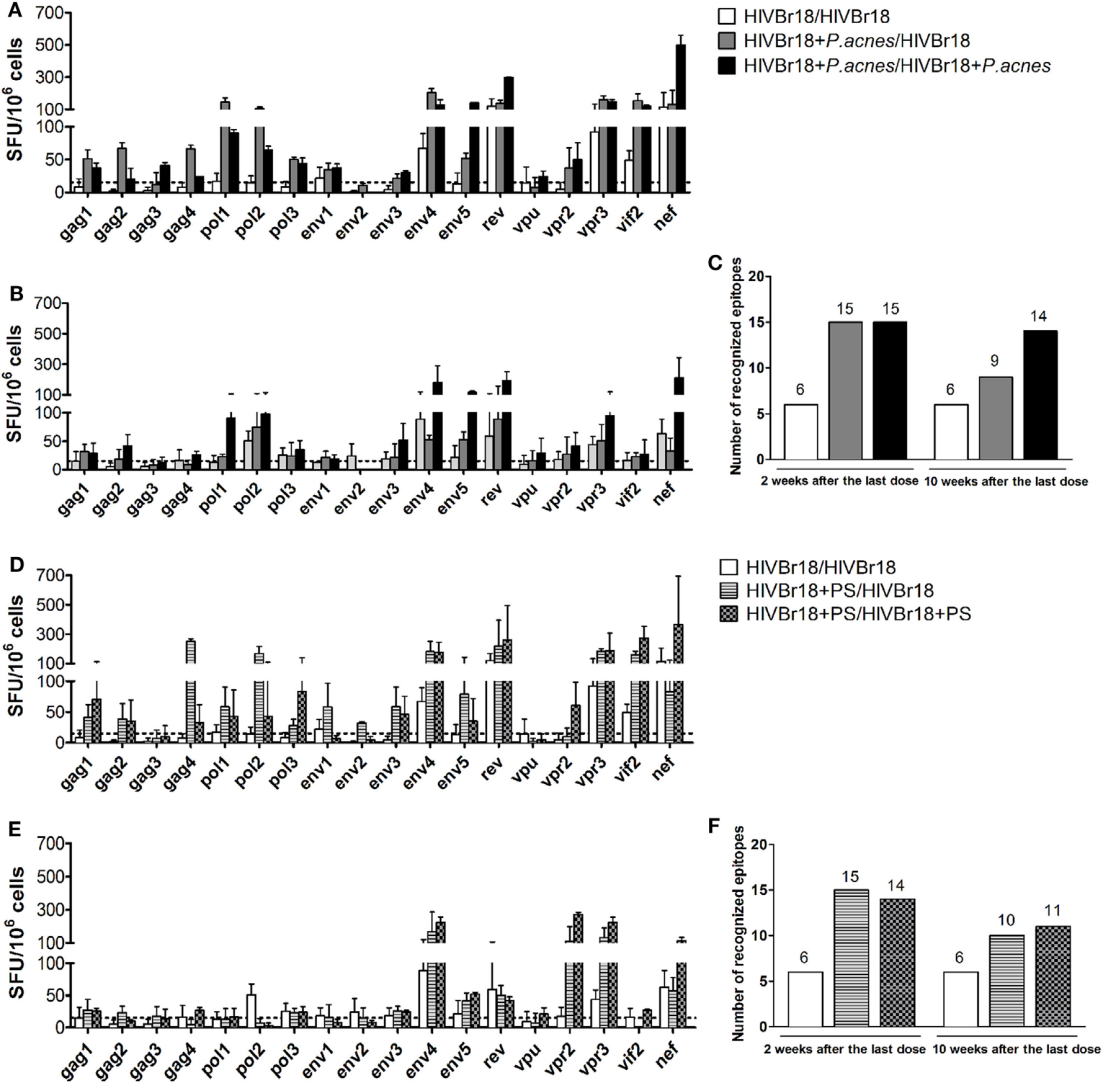
Immunization with HIVBr18 in the presence of *Propionibacterium acnes* or its soluble polysaccharide (PS) fraction increased the magnitude and range of IFN-γ production against the human immunodeficiency virus (HIV)-1 peptides, in a persistent manner. Two or 10 weeks after the last immunization with HIVBr18, in the presence of *P. acnes* [**(A)** for 2 weeks and **(B)** for 10 weeks] or PS [**(D)**, for 2 weeks, and **(E)**, for 10 weeks], spleen cells were separately cultured with each of the 18 peptides for 18 h and evaluated for the magnitude and range of IFN-γ production by ELISpot assay. The number of recognized epitopes **(C,F)** was defined when IFN-γ production was above the cutoff value. SFU, spot-forming units. Cutoff = 15 SFU/10^6^ cells (dotted line). Results are presented as values (mean ± SD) from three independent experiments.

The breadth of the HIV-1-specific cellular response could be another factor indicating the effect of *P. acnes* as an adjuvant on the DNA vaccine HIVBr18 and the respective role of PS in this evaluation. Spleen cells from immunized mice were then separately cultured in the presence of 1 of the 18 peptides encoded by HIVBr18. After 2 weeks, we detected that the breadth of the cellular response was significantly enhanced when HIVBr18 was delivered with *P. acnes* (Figure 2A) or PS (Figure 2D), which was indicated by the average of recognized peptides (Figures 2C,F). Splenocytes from mice immunized with *P. acnes* recognized 15 peptides, and of these 15, mice immunized with PS recognized 14, and mice immunized without adjuvant recognized 6. Moreover, the magnitude of the response to most of the recognized peptides, i.e., SFU value, was higher in the groups that received the adjuvants (Figures 2A,D). Thus, evaluating the HIV-1-specific response *via* IFN-γ production, coadministration of *P. acnes* or PS was able to enhance not only the magnitude but also the range of the response.

Ten weeks after the last dose, only the group that received two doses of the DNA vaccine HIVBr18, both coadministered with *P. acnes*, sustained the closest number (14 peptides) of recognized peptides (Figure 2C). However, groups that received *P. acnes* only in the first dose, or received PS in one or both doses, responded to fewer peptides (9, 10, and 11 peptides respectively, Figures 2C,F). Nevertheless, while the magnitude of positive peptide responses also remained high in both *P. acnes* groups (Figure 2B), this effect was not observed with PS (Figure 2E). Thus, even for the group that received a single dose of *P. acnes* and had a decrease in the number of peptides recognized over time, the magnitude of the positive peptides was still higher than that observed for PS.

### Improvement of the Th1 Response and the Polyfunctional Profile of the Cellular Immune Response After Coadministration of HIVBr18 with *P. acnes* or PS

**Figure 3:**
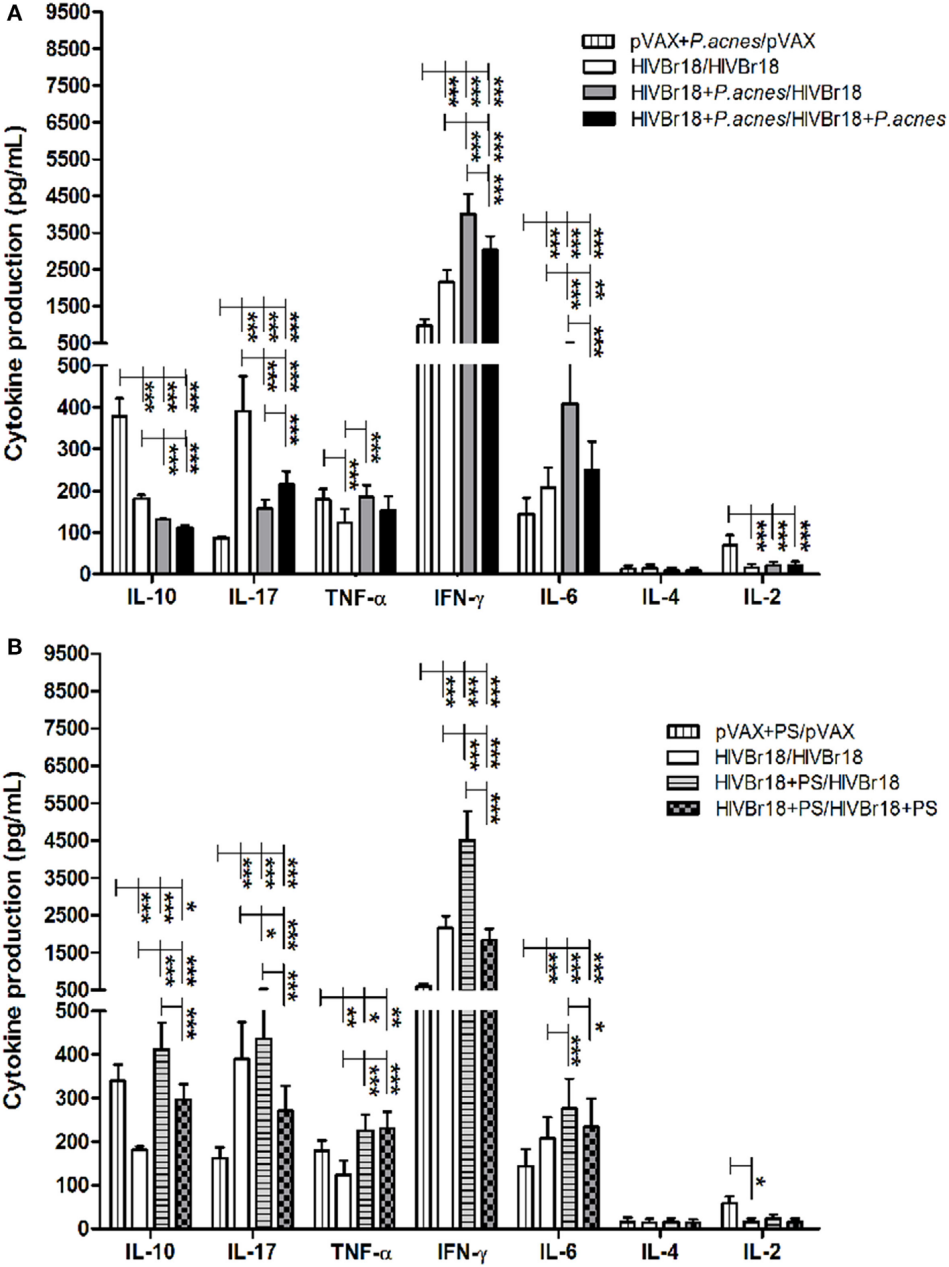
Immunization with HIVBr18 in the presence of *Propionibacterium acnes* or polysaccharide (PS) increased the synthesis of Th1 cytokines against the pool of 18 peptides. Two weeks after immunization with empty vector pVAX1 or HIVBr18, in the presence or absence of *P. acnes* **(A)** or PS **(B)**, splenocytes were cultured for five days with pooled human immunodeficiency virus (HIV)-1 peptides encoded by this DNA vaccine. Cytokines in the supernatant were analyzed using the mouse Th1/Th2/Th17 cytokine cytometric bead array (CBA, BD). Results are presented as values (mean ± SD) from a representative experiment of three independent experiments. *p < 0.05; **p < 0.001; ***p < 0.0001.

Results of the ELISpot assay for IFN-γ revealed that the presence of *P. acnes*, or its PS component, in HIVBr18 immunization was able to induce a driven Th1 response. Thus, splenocyte culture supernatants were evaluated using a cytometric bead array to assess the cytokine profile (Figure 3). The presence of *P. acnes* (Figure 3A) in the first or in both doses generated significantly higher concentrations of IFN-γ (4,012 and 3,027 pg/mL, respectively), IL-6 (408 and 249 pg/mL, respectively), and TNF-α (185 pg/mL, when *P. acnes* was administered in the first dose), than the absence of adjuvant (2,158, 208, and 123 pg/mL, respectively). However, higher concentrations of IL-10 (181 pg/mL) and IL-17 (390 pg/mL) were detected with the absence of adjuvants (control group) than with the groups that received *P. acnes* in the first or in both doses (130 and 112 pg/mL for IL-10, and 157 and 215 pg/mL for IL-17). Levels of IL-4 and IL-2 cytokines were below the detection limit.

Coadministration of PS (Figure 3B) induced higher levels of type I cytokines, such as IFN-γ and TNF-α, than control group. However, compared to the level obtained with *P. acnes* treatment, there was an increase in the levels of IL-10 when PS was administered in the first or in both doses (412 and 298 pg/mL, respectively) and of IL-17 (436 pg/mL) when PS was administered in the first dose.

**Figure 4:**
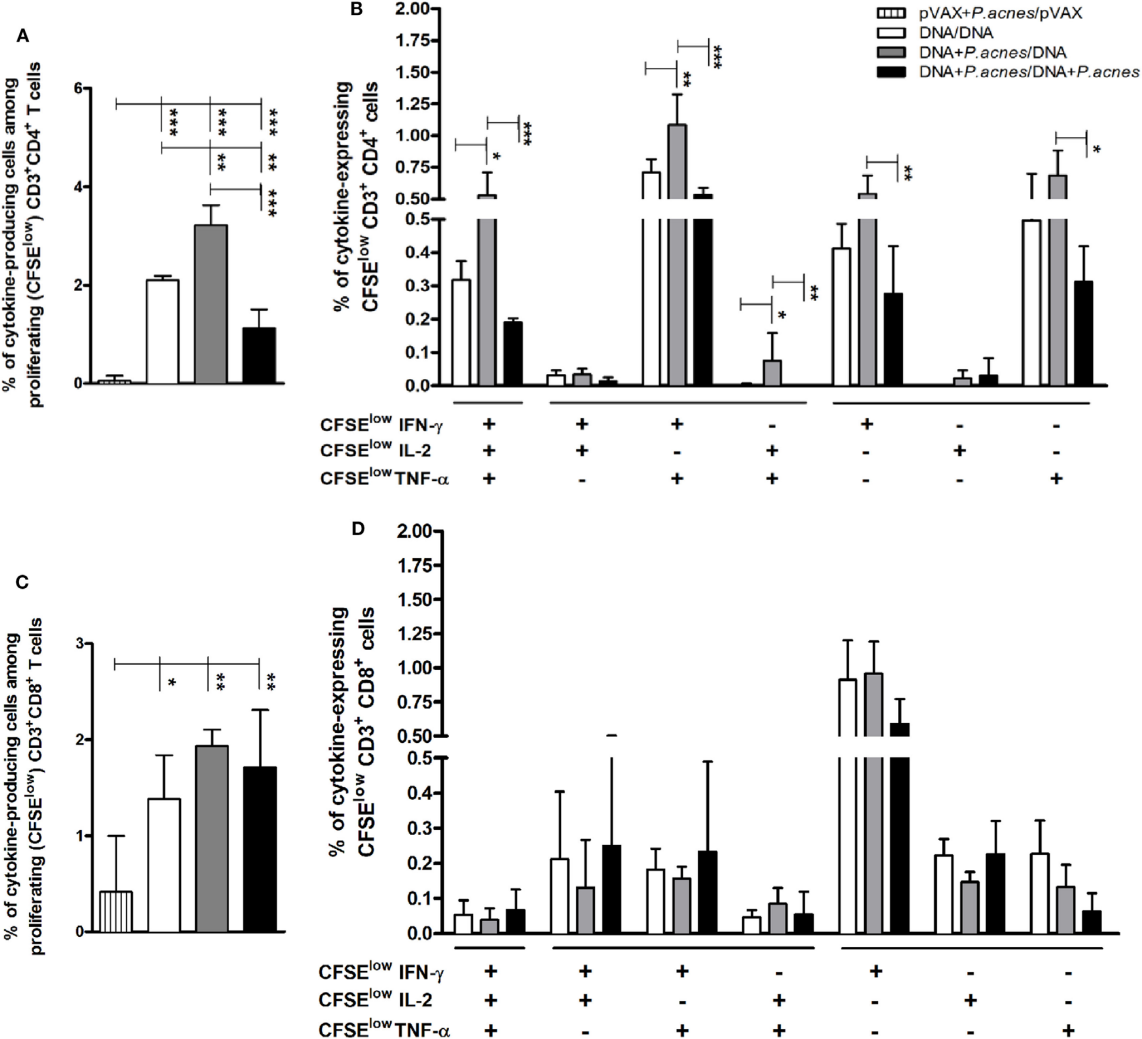
Coadministration of *Propionibacterium acnes* and HIVBr18 vaccine induced an increase in human immunodeficiency virus (HIV)-1-specific CD4^+^ T lymphocytes with a polyfunctional profile of type I cytokines. Two weeks after immunization with empty vector pVAX1 or HIVBr18, in the presence or absence of *P. acnes*, spleen cells were stained with CFSE (1.25 µM) before culturing with pooled HIV-1 peptides for five days. In the last 12 h of incubation, cells were restimulated with Brefeldin A and anti-CD28. After extracellular (CD3, CD4, and CD8) and intracellular staining (IFN-γ, TNF-α, and IL-2), the cells were analyzed by flow cytometry. Panels **(A,C)**, respectively, demonstrate the total frequencies of CD4^+^ and CD8^+^ T lymphocytes, which proliferated and produced cytokines against the stimulus. Panels **(B,D)** demonstrate the Boolean combinations that, respectively, determine the CD4^+^ and CD8^+^ T lymphocytes that proliferated and produced one or more cytokines, using the FlowJo software. Results are presented values (mean ± SD) from three independent experiments. *p < 0.05; **p < 0.001; ***p < 0.0001.

To evaluate whether the adjuvant would affect the phenotypic and functional profile of HIV-1-specific T cells, we used multiparametric flow cytometry. As shown in Figure 4A, administration of *P. acnes* in the first dose of HIVBr18 induced a higher frequency of CD4^+^ T lymphocytes that proliferated and produced any combination of IFN-γ, IL-2, and TNF-α (3.22%) than that induced by administration of HIVBr18 in the absence of adjuvant (2.1%). In addition, Boolean combinations revealed that HIV immunization with *P. acnes* in the first dose increased the frequency of HIV-1-specific polyfunctional CD4^+^ T lymphocytes, as determined by the frequency of double positive IFN-γ^+^/TNF-α^+^ (1.09%) or triple positive (0.51%) cells compared to that in other groups (Figure 4B). In turn, there was no difference in the CD8^+^ T cell population (Figures 4C,D) such that all groups exhibited proliferation of predominantly IFN-γ-producing cells. Similarly, the increase in polyfunctional CD4^+^ T lymphocytes was also observed with coadministration of PS, which increased the frequency of CD8^+^ T lymphocytes that proliferated and produced IFN-γ, IL-2, and TNF-α in any combination (Figure S2 in Supplementary Material).

### Cytotoxic Activity of Splenocytes Induced by Immunization with HIVBr18 in Association to *P. acnes*

As a functional evaluation of immunization with HIVBr18, the ability of splenocytes from immunized mice to induce the lysis of cells that potentially present HIV-1 peptides encoded by this DNA vaccine was investigated. Spleen cells obtained after the last immunization were cultured with a macrophage lineage (RAW 264.7) previously transfected with the HIVBr18 vaccine or with the empty vector and incubated with [methyl-^3^H] thymidine.

**Figure 5:**
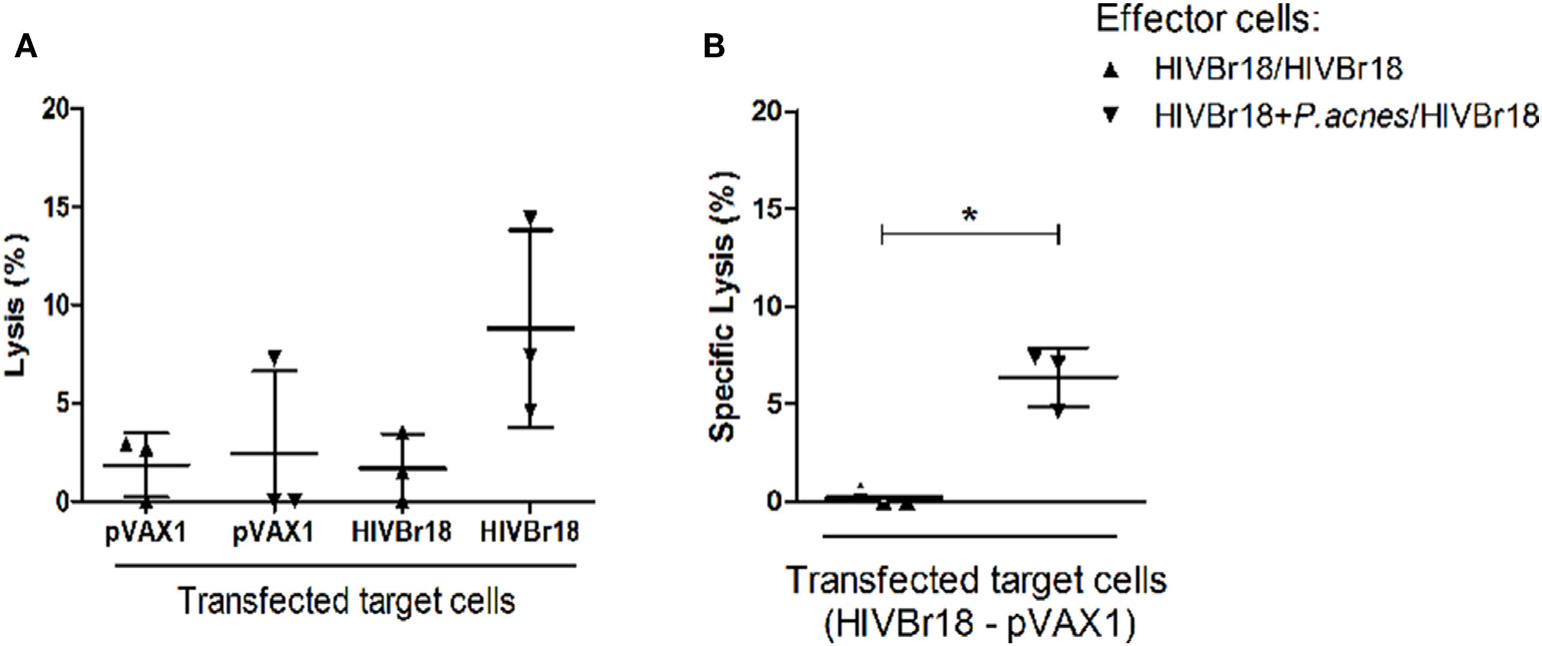
Coadministration of *Propionibacterium acnes* and HIVBr18 DNA vaccine promoted cytotoxic activity of splenocytes against transfected cells. Two weeks after the last immunization with two doses of HIVBr18 alone, or with *P. acnes* in the first dose, spleen cells were obtained and cultured with a macrophage lineage that was previously transfected with the HIVBr18 vaccine plasmid or with empty vector (pVAX1), and then, samples were incubated with [methyl-^3^H] thymidine. Cells were maintained at the proportion of 1 target to 50 effector cells under an atmosphere of 5% CO_2_ at 37°C for 3.5 h. Then, cells were collected in a cell harvester, and the radioactivity was measured in a β-counter and converted to lysis percentage **(A)**. Specific lysis was evaluated by subtracting the lysis value obtained when target cells were transfected with HIVBr18 from the lysis value obtained when target cells were transfected with empty pVAX1 **(B)**. Results are presented as values (mean ± SD) from a representative experiment of two independent experiments. *p < 0.05.

The group that received *P. acnes* was found to be able to induce significant specific lysis (6.37%) when compared to the group immunized with the DNA vaccine alone, which presented undetectable values of specific lysis (Figure 5).

## Discussion

Herein, we have demonstrated the adjuvant effect of heat-killed *P. acnes* suspension, which is known to induce a typical Th1 response, in the immunogenicity of HIVBr18. Specifically, *P. acnes* enhanced the magnitude and range of the response, increasing the number of recognized peptides. Moreover, even with few immunizations, this adjuvant increased the polyfunctional profile of CD4^+^ T cells and prolonged the vaccine-induced immune response. Furthermore, combining the adjuvant *P. acnes* with HIVBr18 immunization induced lymphocyte cytotoxicity to vaccine plasmid-transfected targets.

In this study, reduction in the number of doses used [comparable to the three-dose schedule demonstrated in previous studies (23, 24); Figure S3 in Supplementary Material], and the greater breadth of the response obtained by the coadministration the DNA vaccine and *P. acnes* could be explained by the direct action of this adjuvant on DCs, macrophages and B-2 lymphocytes, i.e., APCs, which has been previously described by us and others (41, 52, 53, 56). A DNA vaccine primes immune responses after intramuscular injection, transfecting mainly myocytes, as well as other immune cells found within the muscle, such as DCs and monocytes (64, 65). Transfection of these cells, or phagocytosis of transfected dead or dying cells by these cells, enables the generation of the immune response through MHC I and/or MHC II pathways, thus priming CTLs and activating CD4^+^ T helper lymphocytes (66–69). Therefore, we believe that the most appropriate adjuvant for DNA vaccine should be the one that potentiates APCs.

Direct effects of *P. acnes* on DC maturation have been described *in vitro* (52–54) and *in vivo*, such that previous studies observed an increase in DC precursors in the liver and blood of mice after treatment with *P. acnes* (70, 71) and an increase in the number and activation status of bone marrow-derived DCs (56). The function of *P. acnes* and the role of its soluble PS component in the activation status of these cells and other APCs have been well investigated. In a murine model of hypersensitivity to ovalbumin (OVA), potentiation of the Th2 response occurred when *P. acnes* or PS treatment coincided with OVA administration, but a typical Th1 response occurred when mice were treated with the adjuvant before OVA sensitization (55, 57). The potentiated OVA-Th2 responses induced by *P. acnes* and PS rely on directed cytokine production, a significant increase in the number of APCs expressing costimulatory molecules, such as CD40 (except PS), CD80, and CD86, and upregulation of these three molecules by mainly macrophages and DCs. However, when Th2 was suppressed, increased expression levels of TLR2, TLR4 and intracellular TLR9 in B cells and DCs and extracellular TLR9 in B cells and macrophages, with increasing numbers of IL-12^+^ cells, were observed. Several studies demonstrated the relationship between TLR2 and TLR4 signaling and the induction of Th1 or suppression of Th2 responses in allergic diseases (72–75). It was also shown that innate immune cells could be directly activated by *P. acnes via* TLR2 and TLR9 (48, 49), thus inducing proinflammatory cytokine synthesis (76). Moreover, *P. acnes* is known to induce production of proinflammatory cytokines, such as IL-18 (77–80), and in combination with IL-12, IL-18 may be responsible for directing the T helper response, resulting in IFN-γ secretion. Therefore, we can speculate that the influence of this adjuvant on APCs—such as improving the cell-mediated immune response (evaluated by IFN-γ production and T cell proliferation), increasing the response magnitude and significantly improving the response range (by increasing the number of recognized peptides)—can explain its impact on the immunogenicity of the HIVBr18 vaccine.

A broad T cell response against conserved epitopes is an essential condition for protection, as demonstrated by T cell-based HIV vaccine trials (20, 81, 82). For example, in the STEP trial, an adenovirus 5-based HIV vaccine encoding three HIV proteins (Gag, Pol, and Nef) induced an average recognition of only three epitopes per participant but in conserved and variable regions of the sequence. This vaccine failed to reduce the viral load in infected individuals and to prevent HIV-1 infection (20, 82, 83). In addition, the induction of simultaneous, vaccine-specific CD4^+^ and CD8^+^ T cell responses was limited to 25% of the patients. However, an adenovirus 5-based SIV vaccine encoding eight SIV proteins promoted a broad CD4^+^ and CD8^+^ T lymphocyte response in vaccinated non-human primates, allowing viremia control after challenge (84).

Unmethylated regions of DNA—such as cytosine-phosphate-guanine oligonucleotide sequences (CpG), which are also common in bacterial genomes—incorporated into DNA vaccines are recognized by TLR9 and induce APC-dependent production of proinflammatory cytokines, i.e., IL-12 and IFN type I, and thus, these regions act as an immune response adjuvant (85, 86). Similarly, *P. acnes* effects are associated with the induction and activation of intracellular and extracellular TLR9 in APCs (49, 58) by inducing the release of Th1 cytokines (87) and IgG2a switch, which is also related to the Th1 pattern (88). This direction to the Th1 pattern was observed both in the Th2 suppression in the type I hypersensitivity murine model described above and using a DNA vaccine containing *T. cruzi* trans-sialidase gene when a higher synthesis of IFN-γ and reduction of IgG1/IgG2a ratio (41) were demonstrated. Indeed, coadministration of HIVBr18 and *P. acnes* increased IFN-γ and TNF-α production but decreased IL-10 production, confirming the Th1 pattern generated by this vaccine (24) and suggesting a greater Th1 polarization upon coadministration of *P. acnes*. Interestingly, immunization with a DNA vaccine in the presence of *P. acnes* led to a decrease in vaccine-specific IL-17 cytokine levels. Moreover, the Th17 response during HIV infection is controversial (89). IL-17A is involved in maintaining the integrity of the epithelial barrier (90, 91), and depletion of gut-associated lymphoid tissue Th17 and Th1/Th17 lymphocytes was shown to be the main cause of chronic immune activation and non-AIDS-related comorbidities in infected individuals (92). However, Th17 cells have been shown to be pathogenic due to their permissiveness to viral infection because they carry integrated HIV-DNA and contribute to persistent reservoirs of HIV under antiretroviral treatment (89, 93).

Intensive investigations of adjuvants that increase the immunogenicity of DNA vaccination have previously demonstrated that coadministration of cytokine genes significantly enhances the immune response (60, 94–97). In a recent work, coadministration of the HIVBr18 DNA vaccine and a plasmid encoding granulocyte-macrophage colony-stimulating factor (GM-CSF) (60) increased the frequency of polyfunctional HIV-specific T lymphocytes (IFN-γ^+^ TNF-α^+^ IL-2^+^). As such, these findings may be related to the increase in these polyfunctional cells observed following coadministration of HIVBr18 and *P. acnes*. A previous study reported an increase in the synthesis of hematopoietic cytokines, such as GM-CSF, in animals treated with *P. acnes* (98), when then induced an increase in the number of bone marrow stem cells and the maturation of DCs (56). HIV-specific and polyfunctional CD4^+^ T lymphocytes were shown to be more frequent in long-term non-progressor patients than in patients who rapidly progressed to AIDS (99, 100). Therefore, the polyfunctional responses of HIV-specific CD4^+^ T cells might be potentiated by the presence of *P. acnes* and might contribute to the control of disease progression (101–106). We also observed higher levels of Th1 cytokines when *P. acnes* was administered in the first dose than when it was administered in both doses. This result could be explained due to cytokine receptor exhaustion or even their delayed turnover.

The levels of IFN-γ-producing cells and proliferating HIV-specific CD4^+^ and CD8^+^ T lymphocytes 10 weeks after the last dose demonstrated that the coadministration of *P. acnes* with both HIV-1 vaccinations is important for longevity of the cellular response. The CD4^+^ T lymphocyte response was previously reported to persist for up to 24 weeks when the DNA vaccine was administered in three doses with a central memory element (24). A vaccine capable of inducing CD4^+^ and CD8^+^ effector memory responses was previously shown to also prevent progressive systemic infection in animals challenged with highly pathogenic SIV, even in the absence of neutralizing antibodies (107). Therapeutic vaccination of monkeys with SIV-specific DNA vaccine with coadministration of IL-12 and IL-15 plasmids enhanced the specific population of CD8^+^ memory cells and their production of cytokines (97). Modulation of both innate and acquired immunity by inducing proinflammatory cytokines, including IL-12, may be one of the mechanisms by which *P. acnes* could modulate the population of memory cells (108), and this mechanism may be associated with the higher frequency of IFN-γ-producing cells and the increased number of recognized peptides.

As expected and as demonstrated in our previous studies, herein, PS is one of the main components of *P. acnes* responsible for the outcomes observed when using the whole bacterium. PS substantially increased the number of cells that specifically produced IFN-γ and increased the amplitude of recognized peptides, the frequency of HIV-specific T lymphocytes (especially the CD8^+^ population), the cytokine pattern (regarding the production of type I cytokines), and the number of CD4^+^ polyfunctional T cells. The presence of other compounds in the whole bacterium (proteins and lipids) could explain the differences observed between *P. acnes* and PS treatments, notably the absence of a significant cognate memory T cell population and a persistent cellular response. However, as demonstrated in previous studies, PS had an undeniable effect in inducing TLR2, TLR4, extracellular and intracellular TLR9 in APCs and B-1 cells (58, 109).

We also found that immunization with the HIVBr18 vaccine, in the presence of *P. acnes*, induced an increased HIV-1-specific cytotoxic activity against target cells transfected with the same plasmid. These target cells could express the majority of epitopes encoded by the DNA vaccine, and herein, the effector cells were found to be able to respond to a large spectrum of epitopes when the adjuvant was coadministered with the vaccine. This activity could be explained by the induction of the Th1 response pattern by the effector pool when *P. acnes* was administered. In addition to these mediators of the adaptive immune response, the adjuvant-potentiated increase in IFN-γ levels could also affect cells with cytotoxic activities, such as macrophages, NK and NKT cells. Published data from our laboratory and others have shown an increased cytotoxic activity of macrophages and NKT cells with high levels of TNF-α and NO release induced by *P. acnes* and its PS fraction (36–38, 110).

A DNA vaccine, such as HIVBr18 vaccine, can induce a potent and persistent response of CD4^+^ T cells with a polyfunctional phenotype and thus can facilitate the cytotoxic activity of these cells. As previously shown in the literature, this type of DNA vaccine may have a protective effect against SIV/HIV infection (15, 18, 19, 22, 84, 99–106). In summary, the adjuvant effect of coadministration of *P. acnes* with HIVBr18 was evidenced by the potentiation of these responses and the increase in the number of recognized peptides, which generated a broader and more persistent response and reduced the number of administered doses. Given the promising use of *P. acnes* in humans, the enhanced responses against conserved HIV-1 regions, such as those observed by coadministering HIVBr18 and *P. acnes*, might control viral replication in individuals who became infected after immunization not only by limiting viral transmission but also by preventing progression to HIV-associated disease.

## Ethics Statement

This study was carried out in strict accordance with the recommendations of the National Institutes of Health Guide for the Care Use of Laboratory Animals and the Brazilian National Law (11.794/2008). The protocol was approved by the Institutional Animal Care and Use Committee (IACUC) of the Federal University of São Paulo and by the Ethics Committee of University of São Paulo School of Medicine (permit number 4836281114).

## Author Contributions

DT and IM conceived and designed the experiments. DT, MI, JA, JV, and VP performed the experiments. DT, MI, JV, and VP analyzed the data and prepared the figures. DT, MI, and IM wrote the manuscript. IM, DR, and EC-N performed the final review of the article. All authors read and approved the final article.

## Conflict of Interest Statement

The authors declare that the research was conducted in the absence of any commercial or financial relationships that could be construed as a potential conflict of interest.
