# Supplementary material for: Propionibacterium acnes Enhances the Immunogenicity of HIVBr18 Human Immunodeficiency Virus-1 Vaccine
Source: Front Immunol. 2018 Feb 7;9:177. doi: 10.3389/fimmu.2018.00177 (PMC5808300; doi:10.3389/fimmu.2018.00177)
Supplement: Supplementary file 1 [file Presentation_1.PDF]

Supplementary Material

PROPIONIBACTERIUM ACNES ENHANCES THE IMMUNOGENICITY OF HIVBr18 HIV-1 VACCINE

Daniela Teixeira, Mayari Eika Ishimura, Juliana de Souza Apostólico, Jacqueline Miyuki Viel, Victor Cabelho Passarelli, Edecio Cunha-Neto, Daniela Santoro Rosa, Ieda Maria Longo-Maugéri*

*** Correspondence:** Corresponding Author: Ieda Maria Longo-Maugéri, imaugeri@unifesp.br

1 Supplementary Figures

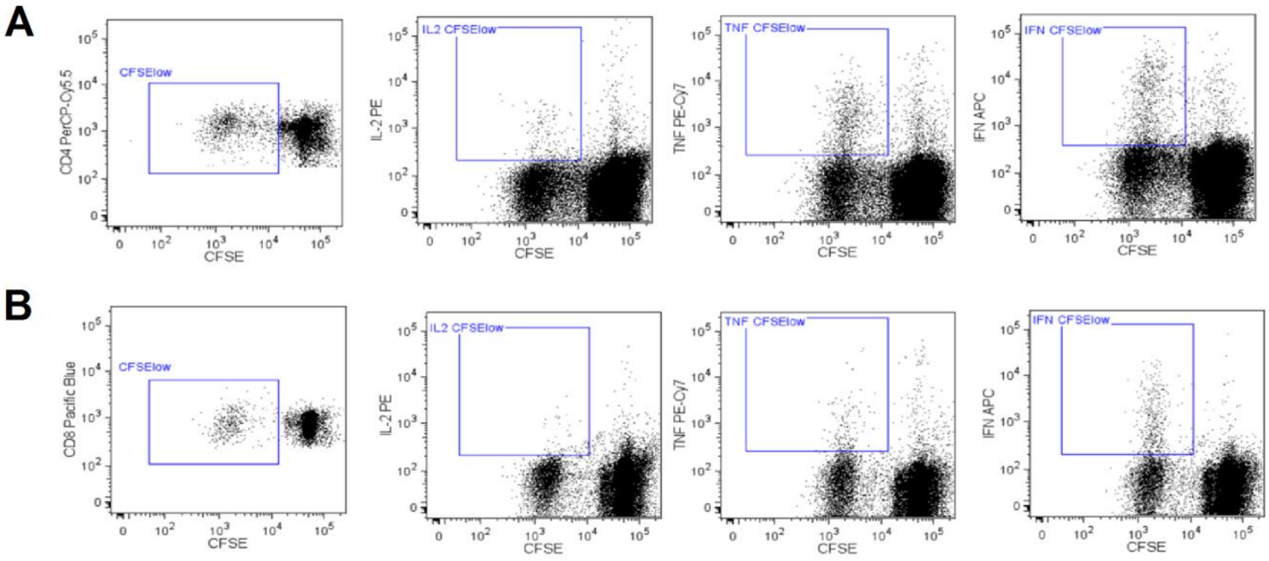

Supplementary Figure 1. Schematic representation of gate strategy to distinguish CD4$^+$ and CD8$^+$ T cells by flow cytometry. BALB/c mice were immunized with two doses of HIVBr18 in the absence or presence of *P. acnes* or PS. Two or ten weeks after the last immunization, spleen cells were (A) labeled with CFSE (1.25 µM) and were either cultured in the presence of the pool of 18 peptides for five days or remained unstimulated. For all analyses, after staining with fluorochrome-conjugated monoclonal antibodies, cells were gated in an FSC x SSC dot plot; after excluding doublets, cells were gated as CD3$^+$ cells and discriminated as CD4$^+$ or CD8$^+$ cells. Antigen-specific proliferation was defined by CFSE dilution in gated CD3$^+$CD4$^+$ (A) and CD3$^+$CD8$^+$ (B) cells. Alternatively, in the last 12 hours of incubation, cells were restimulated in the presence of Brefeldin A and anti-CD28. After labeling for surface molecules, CD3, CD4 and CD8, and then for intracellular IFN-γ, TNF-α and IL-2, gated CD3$^+$CD4$^+$ (A) and CD3$^+$CD8$^+$ (B) cells were analyzed as CFSE^{low} against each intracellular cytokine.

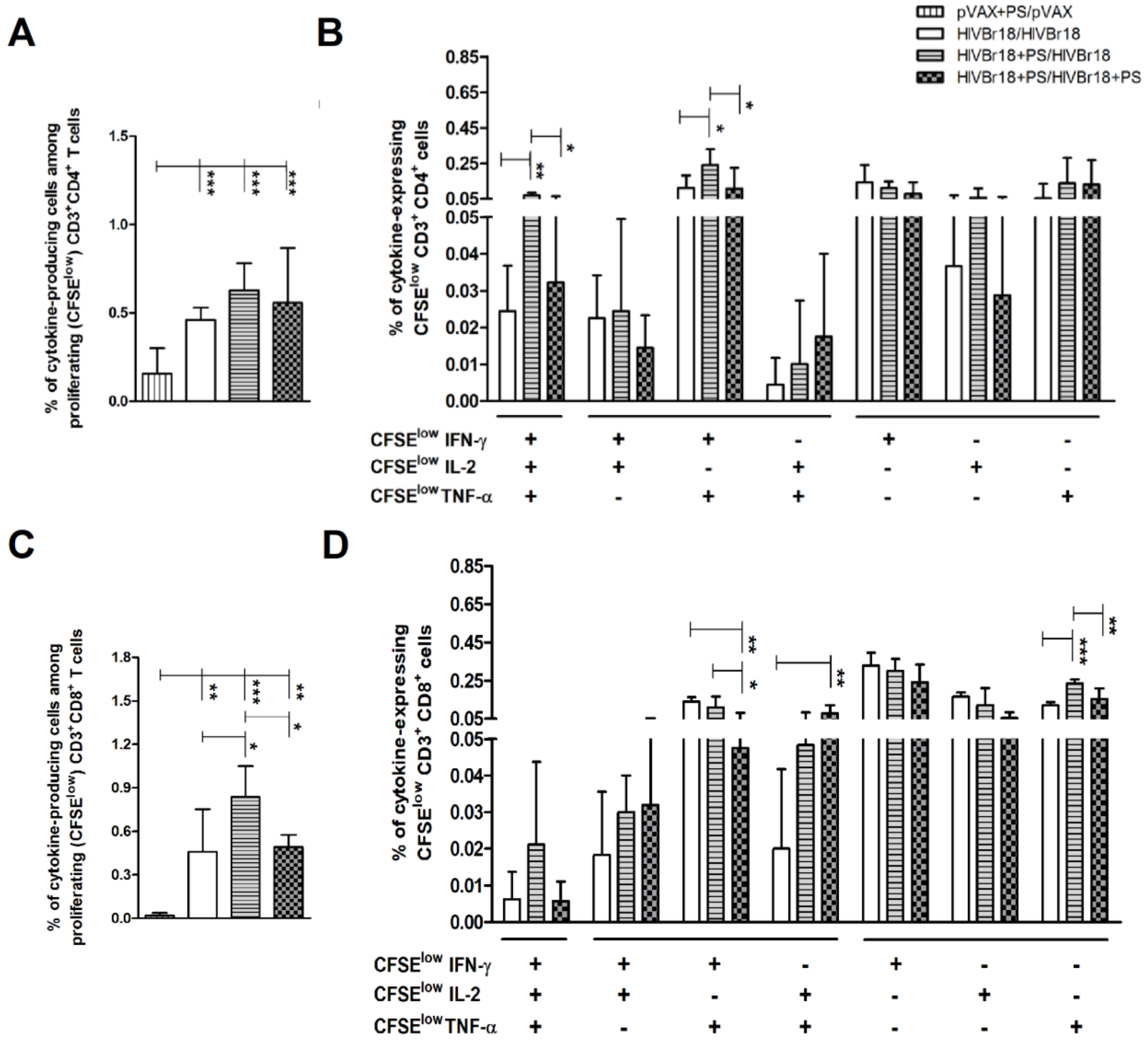

Supplementary Figure 2. Co-administration of PS and HIVBr18 vaccine induced an increase in HIV-1-specific CD4$^+$ T cells with a polyfunctional profile of type I cytokines. Two weeks after the last immunization with empty vector pVAX1 or with HIVBr18, in the presence or absence of PS, spleen cells were labeled with CFSE (1.25 µM) and cultured in the presence of the pool of 18 peptides for five days. In the last 12 hours of incubation, cells were restimulated in the presence of Brefeldin A and anti-CD28. After labeling for surface molecules, CD3, CD4 and CD8 and then for intracellular IFN-γ, TNF-α and IL-2, the cells were analyzed by flow cytometry. Panels A and C respectively demonstrate the total frequencies of CD4$^+$ and CD8$^+$ T lymphocytes, which proliferated and produced cytokines against the stimulus. Panels B and D respectively demonstrate the Boolean combinations that distinguish CD4$^+$ and CD8$^+$ T lymphocytes that proliferated and produced one or more cytokines, using the FlowJo software. Results are presented as values (mean ± SD) from three independent experiments. *p<0.05; **p<0.001; ***p<0.0001.

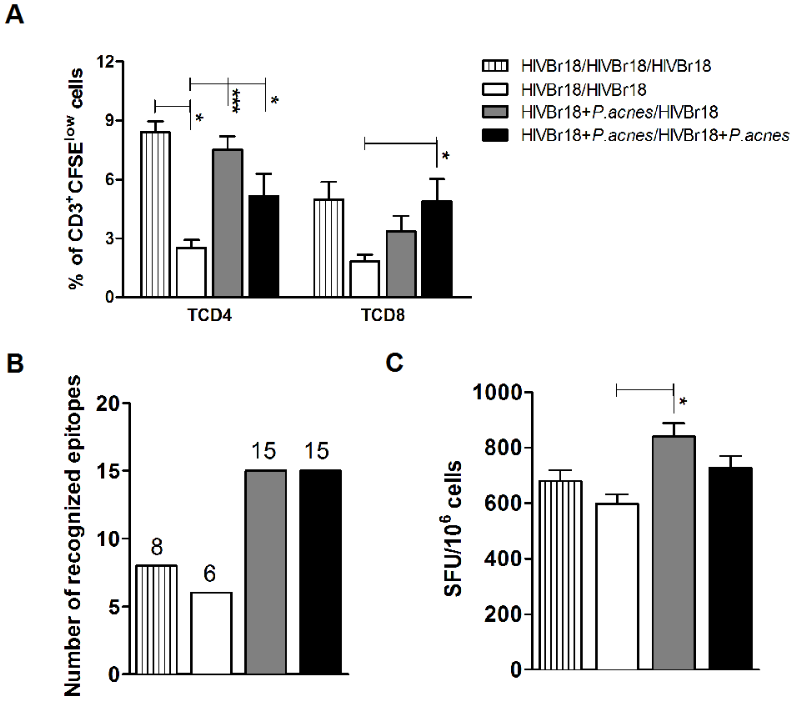

Supplementary Figure 3. Effect of immunization with 2 or 3 doses of HIVBr18, in the presence or absence of *P. acnes*, on the amplitude and magnitude of the cell-mediated immune response. BALB/c mice were immunized with three doses of HIVBr18 or two doses of HIVBr18 in the absence or presence of *P. acnes*. Two weeks after the last immunization, spleen cells were (A) labeled with CFSE (1.25 µM) and cultured in the presence of the pool of 18 peptides for five days. Antigen-specific proliferation was defined by CFSE dilution in gated $CD3^{+}CD4^{+}$ and $CD3^{+}CD8^{+}$ cells by flow cytometry. Alternatively, splenocytes were separately cultured with each of the 18 peptides for 18 hours, and using the ELISpot assay, splenocytes were evaluated for the magnitude of IFN-γ production (defined by the sum of IFN-γ-producing cells for each positive peptide (B)) and the amplitude of this response (defined by the number of recognized epitopes (C)). SFU=Spot-forming units. Cutoff=15 SFU/10^{6} cells. Results are presented as values (mean ± SD) from three independent experiments. *p<0.05; ***p<0.0001.
